# Supplementary material for: Taxonomy-based approach for understanding and enhancing security culture in universities
Source: PeerJ Comput Sci. 2025 Jul 9;11:e3005. doi: 10.7717/peerj-cs.3005 (PMC12453704; doi:10.7717/peerj-cs.3005)
Supplement: Supplemental Information 1 [file peerj-cs-11-3005-s001.docx]

# **Appendix A**

# **List of Primary Studies (FL):**

| **Publication Year** | **Title** | **ID** |
| --- | --- | --- |
| 2010 | M. Kyobe, "Towards a framework to guide compliance with IS security policies and regulations in a university," 2010 Information Security for South Africa, Johannesburg, South Africa, 2010, pp. 1-6, doi: 10.1109/ISSA.2010.5588651.keywords: {Law;Information security;Privacy;Standards;Planning;IS security compliance;security regulations & policies;universities;control frameworks}, | 1 |
| 2011 | Brady, James W. "Securing health care: Assessing factors that affect HIPAA security compliance in academic medical centers." 2011 44th Hawaii International Conference on System Sciences. IEEE, 2011.‏ | 2 |
| 2012 | Maidabino, Abashe Atiku, and A. N. Zainab. "A holistic approach to collection security implementation in university libraries." Library Collections, Acquisitions, and Technical Services 36.3-4 (2012): 107-120.‏ | 3 |
| 2012 | DOLLAH, W., and JUWAHIR ALI. "Determining factors influencing information security culture among ICT librarians." Journal of Theoretical and Applied Information Technology 37.1 (2012).‏ | 4 |
| 2013 | Kortjan, Noluxolo, and Rossouw von Solms. "Cyber security education in developing countries: A South African perspective." e-Infrastructure and e-Services for Developing Countries: 4th International ICST Conference, AFRICOMM 2012, Yaounde, Cameroon, November 12-14, 2012, Revised Selected Papers 4. Springer Berlin Heidelberg, 2013.‏ | 5 |
| 2013 | Furnell, Steven M. "Security education: The challenge beyond the classroom." IFIP World Conference on Information Security Education. Berlin, Heidelberg: Springer Berlin Heidelberg, 2009.‏ | 6 |
| 2013 | Armstrong, Helen. "Two Approaches to Information Security Doctoral Research." IFIP World Conference on Information Security Education. Berlin, Heidelberg: Springer Berlin Heidelberg, 2009.‏ | 7 |
| 2014 | Reid, Rayne, and Johan Van Niekerk. "Towards an Education Campaign for Fostering a Societal, Cyber Security Culture." HAISA. 2014.‏ | 8 |
| 2017 | Hina, Sadaf, and Dhanapal Durai Dominic. "Need for information security policies compliance: A perspective in Higher Education Institutions." 2017 International Conference on Research and Innovation in Information Systems (ICRIIS). IEEE, 2017.‏ | 9 |
| 2017 | Mabece, Thandolwethu, Lynn Futcher, and Kerry-Lynn Thomson. "South African Computing Educators’ Perspectives on Information Security Behaviour." Information Security Education for a Global Digital Society: 10th IFIP WG 11.8 World Conference, WISE 10, Rome, Italy, May 29-31, 2017, Proceedings 10. Springer International Publishing, 2017.‏ | 10 |
| 2018 | Alshare, Khaled A., Peggy L. Lane, and Michael R. Lane. "Information security policy compliance: a higher education case study." Information & Computer Security 26.1 (2018): 91-108.‏ | 11 |
| 2018 | Gustafson, Per. "Modeling the security risk management process in higher education institutions to understand, explain and improve." Advances in Human Factors, Business Management and Leadership: Proceedings of the AHFE 2017 International Conferences on Human Factors in Management and Leadership, and Business Management and Society, July 17− 21, 2017, The Westin Bonaventure Hotel, Los Angeles, California, USA 8. Springer International Publishing, 2018.‏ | 12 |
| 2018 | Ndiege, Joshua RA, and Gabriel O. Okello. "Information security awareness amongst students joining higher academic institutions in developing countries: Evidence from Kenya." The African Journal of Information Systems 10.3 (2018): 4.‏ | 13 |
| 2019 | Nasir, Akhyari, Ruzaini Abdullah Arshah, and Mohd Rashid Ab Hamid. "A dimension-based information security culture model and its relationship with employees’ security behavior: A case study in Malaysian higher educational institutions." Information Security Journal: A Global Perspective 28.3 (2019): 55-80.‏ | 14 |
| 2019 | Kumar, Priya C., et al. "Privacy and security considerations for digital technology use in elementary schools." Proceedings of the 2019 CHI Conference on Human Factors in Computing Systems. 2019.‏ | 15 |
| 2019 | Marchand-Niño, William-Rogelio, and Bruno Paolo Guzman Fonseca. "Social Engineering for Diagnostic the Information Security Culture." 2019 IEEE 39th Central America and Panama Convention (CONCAPAN XXXIX). IEEE, 2019.‏ | 16 |
| 2019 | Ciupercă, Ella Magdalena, Victor VEVERA, and Carmen CÎRNU. "Social Variables of Cyber Security Educational Programmes." eLearning & Software for Education 2 (2019).‏ | 17 |
| 2020 | Lorenz, Birgy, and Kaido Kikkas. "“Trust Me, You Will Need It”: Cybersecurity as Extracurricular Subject at Estonian Schools." International Conference on Human-Computer Interaction. Cham: Springer International Publishing, 2020.‏ | 18 |
| 2020 | Ekpoh, Uduak Imo, Aniefiok Oswald Edet, and Nse Nkereuwem Ukpong. "Security challenges in Universities: Implications for safe school environment." Journal of Educational and Social Research 10.6 (2020): 112-112.‏ | 19 |
| 2020 | Angraini, Rose Alinda Alias, and Okfalisa. "A model of information security policy compliance for public universities: A conceptual model." Emerging Trends in Intelligent Computing and Informatics: Data Science, Intelligent Information Systems and Smart Computing 4. Springer International Publishing, 2020.‏ | 20 |
| 2020 | Drozd, Oleksandr, et al. "Development of ICT models in area of safety education." 2020 IEEE East-West Design & Test Symposium (EWDTS). IEEE, 2020.‏ | 21 |
| 2020 | Kamerilova, Galina S., et al. "Development of Professional Creativity of Teachers in the System of Professional Safety Culture of Children in Transport." The 21st Century from the Positions of Modern Science: Intellectual, Digital and Innovative Aspects. Springer International Publishing, 2020.‏ | 22 |
| 2020 | Nasir, Akhyari, Ruzaini Abdullah Arshah, and Mohd Rashid Ab Hamid. "Information security culture for guiding employee’s security behaviour: A pilot study." 2020 6th International Conference on Information Management (ICIM). IEEE, 2020.‏ | 23 |
| 2020 | Buzdugan, Ar, and Au Buzdugan. "Revision of the curriculum on nuclear safety and security in the light of recent international recommendations." International Conference on Nanotechnologies and Biomedical Engineering. Cham: Springer International Publishing, 2019.‏ | 24 |
| 2020 | Kikkas, Kaido, and Birgy Lorenz. "Training young cybersecurity talents–The case of Estonia." International Conference on Human-Computer Interaction. Cham: Springer International Publishing, 2020.‏ | 25 |
| 2020 | Pavlova, Elitsa. "Enhancing the organisational culture related to cyber security during the university digital transformation." Information & Security 46.3 (2020): 239-249.‏ | 26 |
| 2020 | Nnorom, Stanley U., S. Ezenwagu, and B. C. Nwankwo. "Security management practices in the 21st century for improved university administration." IEE-SEM Journal 8.7 (2020): 1-14.‏ | 27 |
| 2020 | Furnell, Steven, and Nathan Clarke. "Organizational security culture: Embedding security awareness, education, and training." Proceedings of the IFIP TC11 WG 11 (2005): 67-74.‏ | 28 |
| 2020 | Homan, Zenobia, et al. "Developing nuclear security culture at academic and educational institutions." International Journal of Nuclear Security 7.1 (2020).‏ | 29 |
| 2020 | Durojaiye, Tai, Konstantinos Mersinas, and Dawn Watling. "What Influences People’s View of Cyber Security Culture in Higher Education Institutions? An Empirical Study." The Sixth International Conference on Cyber-Technologies and Cyber-Systems. 2020.‏ | 30 |
| 2021 | Ocloo, Charles Mawutor, Adéle Da Veiga, and Jan Kroeze. "A conceptual information security culture framework for higher learning institutions." International Symposium on Human Aspects of Information Security and Assurance. Cham: Springer International Publishing, 2021.‏ | 31 |
| 2021 | Shambabi, Pius Tangeni, Stanford Musarurwa, and Fungai Bhunu Shava. "Assessing Organisational Information Security Culture Among Workforce in Universities: A Case of Namibia." 2021 IST-Africa Conference (IST-Africa). IEEE, 2021.‏ | 32 |
| 2022 | Georgiadou, Anna, Ariadni Michalitsi-Psarrou, and Dimitris Askounis. "Cyber-Security Culture Assessment in Academia: A COVID-19 Study: Applying a Cyber-Security Culture Framework to assess the Academia's resilience and readiness." Proceedings of the 17th International Conference on Availability, Reliability and Security. 2022.‏ | 33 |
| 2022 | Nasir, Akhyari, et al. "Information security culture concept towards information security compliance: a comparison between it and non-IT professionals." International Journal of Integrated Engineering 14.3 (2022): 157-165.‏ | 34 |
| 2022 | Moyo, Moses, et al. "Investigating cyber security awareness among preservice teachers during the COVID-19 pandemic." European, Mediterranean, and Middle Eastern Conference on Information Systems. Cham: Springer International Publishing, 2021.‏ | 35 |
| 2022 | Muhammad, Jika Saidu, et al. "Investigating importance and key factors for information governance implementation in Nigerian Universities." Education and Information Technologies (2022): 1-21.‏ | 36 |
| 2023 | AlKhaza'leh, Mohammad Salman, and Bilal Fayiz Obeid. "THE REALITY OF THE SECURITY CULTURE AT AND THE UNIVERSITY'S ROLE IN PROMOTING IT." Environmental & Social Management Journal/Revista de Gestão Social e Ambiental 17.6 (2023).‏ | 37 |
| 2023 | Arcos-Argudo, Miguel. "Epistemological Justification for the Offer of the Master's Degree in Information Security in Ecuador." International Conference in Information Technology and Education. Singapore: Springer Nature Singapore, 2023.‏ | 38 |
| 2023 | Daneshmandnia, Ali. "Exploring Information Security Processes Effectiveness in Educational Institutions: Impacts of Organizational Factors." 2023 IEEE Asia-Pacific Conference on Computer Science and Data Engineering (CSDE). IEEE, 2023.‏ | 39 |
| 2023 | An, Qin, et al. "How education level influences internet security knowledge, behaviour, and attitude: a comparison among undergraduates, postgraduates and working graduates." International Journal of Information Security 22.2 (2023): 305-317.‏ | 40 |
| 2023 | Marchand-Niño, William-Rogelio, and Yarid-Vanessa Vargas-Malca. "Pretexting and the Information Security Culture. Case of a University of the Peruvian Amazon." 2023 XLIX Latin American Computer Conference (CLEI). IEEE, 2023.‏ | 41 |
| 2023 | Albediwi, Mead Rashed, and Kishwar Sadaf. "A Framework for Cybersecurity Awareness in Saudi Arabia." Journal of Engineering and Applied Sciences 10.1 (2023).‏ | 42 |
| 2023 | Bahtiri, Yllka, et al. "Cyber security in educational institutions." Journal of Natural Sciences and Mathematics of UT 8.15-16 (2023): 307-314.‏ | 43 |
| 2024 | Majjate, Hajar, et al. "Assessing the impact of ethical aspects of recommendation systems on student trust and engagement in E-learning platforms: A multifaceted investigation." *Education and Information Technologies* 30.3 (2025): 3953-3977.‏ | 44 |
| 2024 | Naga, January Febro, and Ma Rowena Caguiat. "Data-Driven Insights for Strengthening Information Security Awareness in Higher Education Institutions." International Conference on Advances in Computational Science and Engineering. Singapore: Springer Nature Singapore, 2023.‏ | 45 |
| 2024 | Estacio, Karen. "Information Security Management in Higher Education Institutions in Compliance with the Organic Law for the Protection of Personal Data." International Conference on Innovation and Research. Cham: Springer Nature Switzerland, 2023.‏ | 46 |
| 2024 | Angafor, Giddeon Njamngang, Iryna Yevseyeva, and Leandros Maglaras. "Securing the remote office: reducing cyber risks to remote working through regular security awareness education campaigns." International Journal of Information Security 23.3 (2024): 1679-1693.‏ | 47 |
| 2024 | Filipenko, Nataliia, et al. "The Concept of Comprehensive Security for Higher Educational Institutions: Ukrainian and European Experience." Conference on Integrated Computer Technologies in Mechanical Engineering–Synergetic Engineering. Cham: Springer Nature Switzerland, 2023.‏ | 48 |
| 2024 | Albinali, Mona, and Mahmood Niazi. "The Security Culture Readiness Model (SCRM) for Saudi Universities: A Preliminary Structure." Proceedings of the 28th International Conference on Evaluation and Assessment in Software Engineering. 2024.‏ | 49 |
| 2024 | Srivastava, Ankit Kumar, et al. "A Framework for Institution to Enhancing Cybersecurity in Higher Education: A Review." LatIA 2 (2024): 14.‏ | 50 |
| 2024 | Mahmood, Samreen, Mehmood Chadhar, and Selena Firmin. "Addressing Cybersecurity Challenges in Times of Crisis: Extending the Sociotechnical Systems Perspective." Applied Sciences 14.24 (2024): 11610.‏ | 51 |
| 2024 | Vincent, Akangbou. "Ethical Leadership and Security Culture in Higher Education." SEAHI Publications, 2024. | 52 |

1. **list of the Grey literature (GL):**

| **ID** | **Name** | **Link** | **Source** |
| --- | --- | --- | --- |
| 1 | A guide to implementing a SAFETY CULTURE in our universities | <https://aplu-prod.s3.amazonaws.com/wp-content/uploads/safety-culture-1.pdf> | Report |
| 2 | Cybersecurity Governance Toolkit | <https://er.educause.edu/articles/2024/1/cybersecurity-governance-toolkit> | Toolkit/Resource |
| 3 | Enhancing Safety and Security in Educational Institutions through Surveillance Systems and Access Control | <https://higherinfogroup.com/enhancing-safety-and-security-in-educational-institutions-through-surveillance-systems-and-access-control/> | Article |
| 4 | The role of the university in strengthening a culture of cyber security | <https://www.univ-alger3.dz/wp-content/uploads/2024/05/Conference-Preamble-EN.pdf> | Report |
| 5 | Fostering a Culture of Security in Higher Education | <https://ventureburn.com/2024/06/fostering-a-culture-of-security-in-higher-education/> | Article |
| 6 | Building a Resilient Cybersecurity Culture in Educational Institutions | <https://secarma.com/building-a-resilient-cybersecurity-culture-in-educational-institutions> | Blog |
| 7 | Why Higher Education Needs to Prioritize Cybersecurity in 2024y Higher Education Needs to Prioritize Cybersecurity in 2024 | <https://www.keepersecurity.com/blog/2024/01/22/why-higher-education-needs-to-prioritize-cybersecurity-in-2024/> | Blog |
| 8 | The State of University Cybersecurity: 3 Major Problems in 2025 | <https://www.upguard.com/blog/top-cybersecurity-problems-for-universities-colleges> | Blog |
| 9 | The Importance of Security in Higher Education Institutions: Strategies and Best Practices | <https://crystalservices.uk.com/the-importance-of-security-in-higher-education-institutions-strategies-and-best-practices/> | Article |
| 10 | Recent FCA Investigations at Universities: A Closer Look at Cybersecurity Compliance | <https://www.womblebonddickinson.com/us/insights/alerts/recent-fca-investigations-universities-closer-look-cybersecurity-compliance> | Article |
| 11 | ENHANCING NETWORK SECURITY IN ACADEMIC INSTITUTIONS THROUGH USER EDUCATION AND AWARENESS PROGRAMS: ADDRESSING VULNERABILITIES AND PROMOTING BEST PRACTICES | <https://www.ijramr.com/sites/default/files/issues-pdf/5345_0.pdf> | Article |
| 12 | Privacy by Design: Building a Data Protection Culture in Higher Education Institutions | <https://medium.com/@andraylawrence261/privacy-by-design-building-a-data-protection-culture-in-higher-education-institution-2a3abb9c263d> | Blog |
| 13 | How Higher Education Institutions Can Strengthen Their Cyber Resilience | <https://www.linkedin.com/pulse/how-higher-education-institutions-can-strengthen-cyber-tejus-kothari/> | Blog |
| 14 | How to Improve Cybersecurity Awareness in Educational Institutions | <https://cfisa.com/how-to-improve-cybersecurity-awareness-in-educational-institutions/> | Article |
| 15 | The Impact of Security Breaches on Educational Institutions | <https://www.bravurasecurity.com/blog/the-impact-of-security-breaches-on-educational-institutions> | Blog |
| 16 | A Recap of Recent Cybersecurity Incidents at Universities | <https://www.schellman.com/blog/cybersecurity/cybersecurity-incidents-at-universities-2023> | Blog |
| 17 | Cybersecurity threats in educational institutions | <https://preyproject.com/blog/cyber-security-threats-it-professionals-in-education-face> | Blog |
| 18 | The Role of Cybersecurity in Schools and Universities | <https://www.linkedin.com/pulse/role-cybersecurity-schools-universities-strongbox-it-pvt-ltd-jpdte/> | Blog |
| 19 | Educational Institutions: How to Ensure Data Compliance and Security | <https://www.endpointprotector.com/blog/educational-institutions-how-to-ensure-data-compliance-and-security/> | Blog |
| 20 | The Importance of Safety Culture in Higher Education | <https://www.centegix.com/blog/the-importance-of-safety-culture-in-higher-education/> | Bolg |
| 21 | The Importance of Building a Security Culture in Educational Institutions \| Safeguard Security | <https://www.safeguardcalgary.com/blogs/the-importance-of-building-a-security-culture-in-educational-institutions-safeguard-security/> | Blog |
| 22 | Developing a cybersecurity strategy for higher education institutions | <https://www.6clicks.com/resources/blog/developing-a-cybersecurity-strategy-for-higher-education-institutions> | Blog |
| 23 | Education sector: why cyber security awareness needs to be a priority | <https://thesecuritycompany.com/the-insider/education-sector-cyber-security-awareness-needs-to-be-a-priority/> | Blog |
| 24 | Creating Safety Cultures in aCademic Institutions: | <https://www.acs.org/content/dam/acsorg/about/governance/committees/chemicalsafety/academic-safety-culture-report.pdf> | Report |
| 25 | A Campus Culture of Cybersecurity How to teach your faculty, students, and staff to be more secure | <https://focus.vpfinance.virginia.edu/sites/focus.vpfinance/files/2019-11/Campus%20Culture%20of%20Cybersecurity.pdf> | Report |
| 26 | THE CYBER THREAT TO EDUCATION AND ACADEMY TRUSTS IN THE UK | <https://mooreks.co.uk/wp-content/uploads/2022/10/Cyber-Threat-to-Education.pdf?utm_source=Insight&utm_medium=Insight+download&utm_campaign=Cyber-Threat-to-Education> | Report |
| 27 | Prioritizing Security in Higher Education | <https://evolllution.com/technology/security_compliance/prioritizing-security-in-higher-education> | Article |
| 28 | Cybersecurity in education: Why is it important and how can UEBA help? | <https://www.manageengine.com/log-management/cyber-security/cybersecurity-in-education-importance-ueba.html> | Article |
| 29 | Information security for higher education Tools and practices for identifying, assessing, and managing risk | <https://www.ellucian.com/assets/en/ebook/information-security-higher-education.pdf> | Whitepaper |

# 
