# Supplementary material for: Taxonomy-based approach for understanding and enhancing security culture in universities
Source: PeerJ Comput Sci. 2025 Jul 9;11:e3005. doi: 10.7717/peerj-cs.3005 (PMC12453704; doi:10.7717/peerj-cs.3005)
Supplement: Supplemental Information 2 [file peerj-cs-11-3005-s002.docx]

Table 11 List of best practices.

| **No.** | **Best Practices** |
| --- | --- |
| 1.1.1 | A system has been established to deploy a two-factor or multi-authentication security system across the organization for all employees to enhance account protection. |
| 1.1.2 | IT departments are asked to classify data according to its sensitivity and establish appropriate handling guidelines. |
| 1.1.3 | The university has set procedures or rules requiring IT departments to consistently review and update user access privileges to ensure they align with job responsibilities. |
| 1.2.1 | Employees are asked to avoid visiting unknown websites or downloading software from untrusted sources. |
| 1.2.2 | Awareness seminars have emphasized the importance of employees not downloading anything from nonwork-related websites. |
| 1.2.3 | Implement secure configurations for web servers. |
| 1.3.1 | The university has established procedures or rules directing individuals to use the university VPN when appropriate and necessary to securely connect to the university network. |
| 1.3.2 | Use two-factor authentication for VPN access. |
| 1.3.3 | Employ strong authentication mechanisms for Wi-Fi access (WPA2- WPA3). |
| 1.4.1 | A system will automatically log out an employee if the computer is not used for 30 minutes. |
| 1.4.2 | Employees are asked to lock Windows every time they leave their workstations unattended. Press CTRL-ALT-DEL simultaneously. |
| 1.4.3 | Disable or delete stuff accounts to prevent unauthorized access when leaving the university. |
| 1.5.1 | The university has a policy in place that requires the use of licensed software across campus. |
| 1.5.2 | Employees are asked to ensure antivirus software is up to date and make sure their antivirus is set to automatically receive updates. |
| 1.5.3 | The university has a policy in place to ensure that all computers on campus have up-to-date antivirus software. |
| 1.5.4 | Employees are asked to establish robust data backup and recovery processes to ensure minimal data loss in case of a security incident. |
| 1.5.5 | Employees are asked to enable computer firewalls. |
| 1.5.6 | The organizational units have documented policies and control mechanisms in place to regularly back up their data and encrypt these backups for security purposes. |
| 1.5.7 | A system has been established to ensure automatic updates for the operating system are consistently turned on. |
| 1.5.8 | The university has a policy in place to ensure that all computers on campus have antivirus software installed. |
| 1.5.9 | Employees are asked to prevent the installation of unlicensed/unapproved software. |
| 1.5.10 | Awareness seminars have emphasized the use of web browsers such as Chrome or Firefox that receive regular, automatic security updates among employees. |
| 1.6.1 | Sufficient awareness has been raised regarding the importance of employees not opening emails (especially attachments) from unknown or unexpected senders. |
| 1.6.2 | Employees are asked to use a non-University email address for personal email. |
| 1.6.3 | A system has been created to report spam or suspicious messages to the IT center. |
| 1.6.4 | Sufficient awareness has been created that an employee should not respond to any email requesting confidential information (username, password, Social Security number, etc.). |
| 1.6.5 | The university maintains a policy prohibiting employees from downloading unauthorized documents or software. |
| 1.7.1 | Employees are asked to choose a strong password, one that contains numbers, letters (uppercase and lowercase), and special characters and is at least eight characters long. |
| 1.7.2 | The university has the policy to change default passwords on systems or devices where possible (with what frequency or time limit [i.e., monthly]). |
| 1.7.3 | The university has a procedure or rules that restrict the reuse of strong passwords across various devices, servers, software, or applications. Typically, it mandates a specific number of previous passwords that cannot be reused. |
| 1.7.4 | Organizational units have documented policies and control mechanisms ensuring that employees do not write passwords down or store them anywhere in their office or a file of any computer system without encryption. |
| 2.1 | Awareness seminars have emphasized the importance for the university to conduct regular information security training for all staff, students, and faculty members. |
| 2.2 | The IT or cybersecurity department is asked to make online security articles on the computer screen savers for all employees (that change every month). |
| 2.3 | The university has established procedures or rules aimed at developing engaging training materials and resources to educate the university community about security best practices and potential threats. |
| 2.4 | The university has a policy in place to organize special security events and days on campus. |
| 2.5 | The human resources department is asked to conduct employee orientation programs to familiarize new employees with university policies, culture, benefits, and their roles within the institution. |
| 3.1 | The IT department or the university’s training and development department is asked to conduct regular training sessions to educate employees about various security threats. |
| 3.2 | The training and development department or IT department is asked to engage employees through interactive workshops, quizzes, and real-life examples to make the training sessions more relatable and impactful. |
| 3.3 | Awareness seminars have emphasized the importance of the university implementing a reward system for employees who identify and report potential threats such as suspicious emails. |
| 3.4 | Sufficient awareness has been created that the university should provide security awareness training on recognizing and reporting potential indicators of insider threats. |
| 3.5 | The IT department and the training and development team are asked to train students, faculty, and staff on general data security, cybersecurity threats, how to recognize threats, and what to do if a threat occurs. |
| 3.6 | Awareness seminars have highlighted the importance of the university ensuring that all users complete Security Awareness Training sessions. |
| 3.7 | The cybersecurity team or individuals within the IT department are asked to use straightforward, concise language to explain the security threats. |
| 3.8 | The university has a policy allowing the IT department to conduct training for employees on privacy and security issues. |
| 3.9 | The training and development unit or IT department is asked to create high-quality training materials. |
| 4.1 | Organizational units have policies and control mechanisms in place that require top management to schedule regular meetings with other teams. |
| 4.2 | The university’s top management should promote both informal and formal daily communication among team members. |
| 4.3 | The department heads are asked to assign qualified employees at every level for improved process output. |
| 5.1 | Awareness seminars have highlighted the importance of educating staff about threats and what the aviation sector has in place to counter them. |
| 5.2 | The university has a policy to teach staff how to properly manage and move data within its appropriate environment to minimize possible data exposure. |
| 5.3 | IT departments are asked to seek feedback and input from students, faculty, and staff to improve security measures. |
| 6.1 | Transparency in data handling—clearly communicate how data is collected, stored, and used within the university. |
| 6.2 | Data privacy measures/implement robust data privacy measures in accordance with relevant regulations (e.g., GDPR, CCPA) to protect personal information from unauthorized access or misuse. |
| 6.3 | Protect the university’s reputation and ability to ensure business continuity. |
| 7.1 | The university has established procedures or rules instructing the IT department and the risk management team to identify a disaster recovery plan. |
| 7.2 | The IT department and risk management team are asked to develop and implement a risk mitigation plan resulting from the evaluation. |
| 7.3 | Awareness seminars have emphasized the importance of installing emergency communication systems that can quickly and effectively alert students, faculty, and staff in the event of an emergency. |
| 7.4 | The IT department and risk management team are asked to develop a risk management program to identify and assess potential risks and vulnerabilities and prioritize resources accordingly. |
| 7.5 | The university has a policy in place for the IT department and the risk management team to conduct regular security assessments, penetration testing, and vulnerability scans. |
| 7.6 | The IT department and risk management team are asked to evaluate risks from third-party components. |
| 7.7 | Organizational units have documented policies and control mechanisms that let the IT department and risk management team evaluate current risks once a week and discuss potential new ones. |
| 8.1 | Ensure that the university stay informed about relevant data protection and privacy laws and ensure compliance with them. |
| 8.2 | Maintain a culture of continuous improvement and adaptability to changing regulatory requirements. |
| 8.3 | The university has a policy in place for the IT department and risk management team to identify the information security consequence. |
| 9.1 | Employees are asked to avoid engaging in cheating, lying, fraud, theft, extortion, or other dishonest behaviors systematically within HEIs. |
| 9.2 | The university has a policy for employees, requiring them to demonstrate behavior consistent with a set of positive moral and ethical principles and standards, grounded in core values, among education actors. |
| 9.3 | The university has a policy for employees, requiring them to treat others with impartiality, ensuring freedom from discrimination or dishonesty within HEIs. |
| 10.1 | Sufficient awareness has been created that any employee does not transport sensitive data on devices such as laptops or memory sticks that can be lost or stolen. |
| 10.2 | A system has been created to prevent unauthorized and unintended information transfer via shared system resources. |
| 10.3 | A system has been designed to encrypt communications and sensitive data stored on campus servers or networks. |
| 11.1 | Establish a well-defined and documented change management process that outlines the steps, roles, and responsibilities involved in making changes to the university’s systems and infrastructure. |
| 11.2 | Implement a robust change authorization mechanism that ensures changes are reviewed and approved by authorized personnel before implementation. |
| 11.3 | Maintain comprehensive documentation for all changes made to systems and infrastructure. |
| 12.1 | Employees are asked to behave securely through monitoring and control, reward and deterrence, and applicability. |
| 12.2 | IT departments are asked to make sure that employees handle password management and phishing incidents in a secure manner. |
| 12.3 | Teach data privacy and ethics courses to encourage students to think critically about attitudes toward privacy. |
